# Structure and Function of Cochlear Gap Junctions and Implications for the Translation of Cochlear Gene Therapies

**DOI:** 10.3389/fncel.2019.00529

**Published:** 2019-11-27

**Authors:** Xuewen Wu, Wenjuan Zhang, Yihui Li, Xi Lin

**Affiliations:** ^1^Department of Otolaryngology, Head-Neck and Surgery, Xiangya Hospital of Central South University, Changsha, China; ^2^Department of Otolaryngology, Emory University School of Medicine, Atlanta, GA, United States; ^3^Department of Otolaryngology, Wuhan Union Hospital, Tongji Medical College, Huazhong University of Science and Technology, Wuhan, China; ^4^Department of Pharmacy, Changsha Hospital of Traditional Medicine, Changsha, China

**Keywords:** gap junction, connexin, cochlea, structure and function, gene therapy, deafness

## Abstract

Connexins (Cxs) are ubiquitous membrane proteins that are found throughout vertebrate organs, acting as building blocks of the gap junctions (GJs) known to play vital roles in the normal function of many organs. Mutations in Cx genes (particularly *GJB2*, which encodes Cx26) cause approximately half of all cases of congenital hearing loss in newborns. Great progress has been made in understanding GJ function and the molecular mechanisms for the role of Cxs in the cochlea. Data reveal that multiple types of Cxs work together to ensure normal development and function of the cochlea. These findings include many aspects not proposed in the classic K^+^ recycling theory, such as the formation of normal cochlear morphology (e.g., the opening of the tunnel of Corti), the fine-tuning of the innervation of nerve fibers to the hair cells (HCs), the maturation of the ribbon synapses, and the initiation of the endocochlear potential (EP). New data, especially those collected from targeted modification of major Cx genes in the mouse cochlea, have demonstrated that Cx26 plays an essential role in the postnatal maturation of the cochlea. Studies also show that Cx26 and Cx30 assume very different roles in the EP generation, given that only Cx26 is required for normal hearing. This article will review our current understanding of the molecular structure, cellular distribution, and major functions of cochlear GJs. Potential implications of the knowledge of cochlear GJs on the design and implementation of translational studies of cochlear gene therapies for Cx mutations are also discussed.

## Introduction

Connexins (Cxs) are ubiquitous membrane proteins that are present throughout the vertebrate organs. Six Cx subunits are assembled into a connexon, a hexameric structure in the cell membrane (also called a hemichannel; Dermietzel et al., 1990; Liu et al., 2006; Laird and Lampe, 2018). Undocked hemichannels provide conduits to connect intracellular and extracellular spaces when opened upon different stimuli and allow movement of molecules such as ions, ATP and fluorescent dyes (Alstrøm et al., 2015). Two hemichannels align to form one gap junction (GJ) channel that spans the plasma membrane and provides a conduit connecting the intracellular spaces of two adjacent cells (Dermietzel et al., 1990). These intercellular channels facilitate the movement of ions and biochemically-active molecules [e.g., glucose (Chang et al., 2008), ATP (Bevans and Harris, 1999; Anselmi et al., 2008), miRNA (Zong et al., 2016), and cell signaling molecules such as second messengers (Laird and Lampe, 2018)] that are essential for nutritional and signaling needs of cells. Invertebrates have similar proteins called innexins (Phelan et al., 1998; Güiza et al., 2018; Slivko-Koltchik et al., 2019). Another family of proteins shared between lower chordates and vertebrates are termed pannexins (Baranova et al., 2004; Tang et al., 2008), which generally form membrane channels that connect the intracellular and extracellular spaces, but rarely form GJs (Beyer and Berthoud, 2018). Currently there are 21 and 20 subtypes of Cxs found in humans and mice, respectively. Nineteen of these Cxs are orthologs that are shared between the two species (Söhl et al., 2003; Bedner et al., 2012). Compatible Cx subtypes co-assemble to form GJs in order to perform specific functions appropriate for local microenvironment (White, 2002; Ahmad et al., 2003). The most common GJs found in the adult rodent cochlea are co-assembled from Cx26 and Cx30, both of which are present in all types of non-sensory cells in the cochlea (Ahmad et al., 2003; Sun et al., 2005; Hoang Dinh et al., 2009).

GJs play vital roles in maintaining homeostasis of the microenvironment of cell-cell interactions in tissues (Zhao et al., 2006; Meşe et al., 2007). These intercellular channels are essential in the normal development and function of many organs, especially where microcirculation is poor, such as lens in the eyes (White, 2002) and the organ of Corti in the cochlea (Wang Y. et al., 2009) where physiology requirements demand special arrangements. The formation of morphogen gradients during development is critically dependent on GJs (Pietak and Levin, 2018). During cardiovascular and uterine muscle development, synchronization of myocyte contraction depends on the transmission of action potentials through GJ-mediated “electrical synapses” (Delorme et al., 1997). Mutations in various types of Cxs directly cause a large spectrum of human diseases (Srinivas et al., 2018). Most relevant to this review, mutations in Cxs cause more than half of all congenital cases of both syndromic and non-syndromic deafness (Hoang Dinh et al., 2009; Beheshtian et al., 2016).

**Figure 1:**
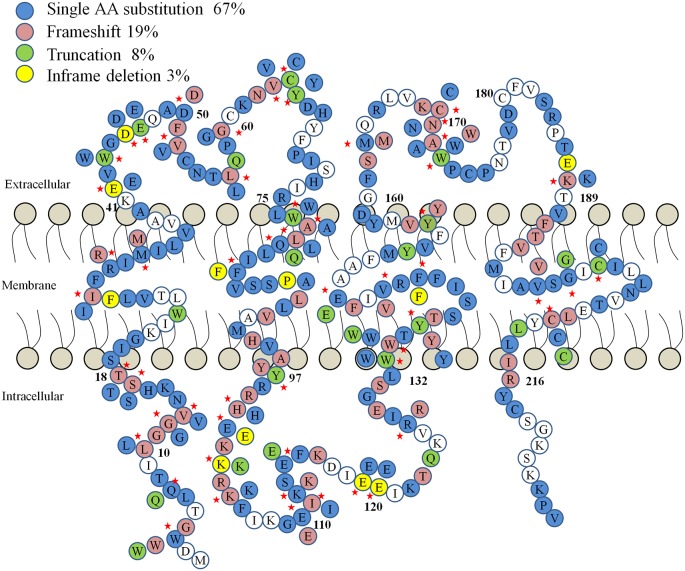
A diagram of human Connexin 26 (Cx26) protein mutations linked to hearing loss. The four types of mutations (frame shift, truncation, single amino acid substitution and in-frame deletion) are color-coded. Red stars denote residues with more than one type of mutation.

The idea of GJs in the organ of Corti was first suggested in the 1970s, when ultrastructural examination of cochlear morphology led to the proposal of a “functional syncytium” among supporting cells in the organ of Corti (Jahnke, 1975; Iurato et al., 1977). The essential role of GJs in hearing is revealed through numerous genetic linkage analyses and functional studies associating mutations in Cx genes with congenital hearing impairment in humans (White et al., 1998; Bruzzone et al., 2003; Chang et al., 2003; Del Castillo et al., 2003). Meanwhile, various roles for hemichannels in cochlear supporting cells and lateral wall cells have been proposed as well, such as releasing of K^+^, ATP or IP3 into extracellular space by cochlear cells (Verselis, 2019). Abnormal opening of hemichannels caused by *GJB2* mutations has serious consequences on the ability of cells to maintain homeostasis and are suspected to cause syndromic hearing loss that may lead to death in early childhood (Stong et al., 2006). The accumulation of data demonstrates that mutations in *GJB2*, which encodes Cx26, account for approximately half of all inherited prelingual non-syndromic deafness cases in both European and East Asian populations (Maw et al., 1995; Morell et al., 1998; Dai et al., 2009, 2015; Liu X. Z. et al., 2009). It is now clear that mutations in Cx genes are one of the most common genetic causes of hearing loss. Recently, a role for Cx26 in noise- and age-dependent hearing loss has also been proposed (Wang et al., 2014; Wu et al., 2014; Zhou et al., 2016; Zong et al., 2017). In addition, hundreds of mutations associated with human deafness have been identified in Cxs other than *GJB2*, such as* GJB6* (encoding Cx30; Grifa et al., 1999), *GJB3* (encoding Cx31; Xia et al., 1998; López-Bigas et al., 2001)*, GJE1* (encoding Cx29; Yang et al., 2007), and *GJB1* (encoding Cx32; Rabionet et al., 2000,^1^). The deafness-linked* GJB2* mutations include at least 93 truncation mutations, 239 point mutations that are known to cause either non-syndromic or syndromic deafness. Most of these mutations are inherited in the autosomal recessive manner, but there are at least 14 reported autosomal dominant point-mutations as well (Figure 1).

A complex set of Cxs forming homotypic/heterotypic/heteromeric GJs are expressed in the cochlea in developmentally-regulated and cell type-specific manners. Recent data suggest that Cx26 and other Cxs play essential roles in the development and maintenance of the cochlear function (Ahmad et al., 2003; Qu et al., 2012; Chang et al., 2015a; Mammano, 2018), such as the formation of the normal cochlear morphology, development of the endocochlear potential (EP; Qu et al., 2012; Wingard and Zhao, 2015; Mammano, 2018). In contrast to the assumptions made by the K^+^ recycling theory, Cx mutations clearly affect GJ function long before the establishment of the high concentration (~150 mM) of K^+^, the EP, and the onset of hearing (Kamiya et al., 2014). Many studies have suggested plausible theories (e.g., expression profile, functional maturation of the cochlea, hair cells (HCs) synaptic formation, etc.; Chang et al., 2015a; Jagger and Forge, 2015; Zhao, 2017). This review article will provide a brief review of our current understanding of the molecular structure, cellular distribution and function of cochlear GJs, as well as the translational relevance of knowledge of cochlear GJs to the success of cochlear gene therapies for Cx mutations.

## Molecular Structure and Pattern of Expression of Cochlear GJs

### General Protein Structure of Cochlear GJs

Most mammalian Cx genes consist of two exons, with exon 1 encoding the 5′-untranslated region (5′-UTR), while exon two contains the complete coding sequence (CDS) and the 3′-UTR. However, *GJB1* and *GJC1* (encoding Cx32 and Cx45, respectively) have more than two exons, with exon 3 encoding the CDS (Teunissen et al., 2007). Hydropathy plots of Cxs predict a common membrane topology consisting of four hydrophobic membrane-spanning domains (M1–M4), connected by two extracellular loops (El and E2) and one intracellular loop, with C- and N-termini both in the cytoplasm. The N-terminus and extracellular loops appear to be relatively conserved across paralogous Cxs, while the C-terminus shows the most variability. The C-terminus is not essential for surface expression of Cxs, as truncating mutations do not prevent trafficking to the membrane or GJ assembly (Martin et al., 2000). However, the C-terminus is the major site for phosphorylation and contains sites for interacting with non-Cx proteins (e.g., cell junction or cytoskeleton proteins) such as vinculin, calmodulin, ZO-1, and spectrin (Lampe and Lau, 2000; Batissoco et al., 2018). These findings suggest that variability in the C-terminus may affect the trafficking of Cxs. In contrast, the N-terminus of Cxs has been found to form part of the pore entrance (Purnick et al., 2000; Musa et al., 2004) together with the transmembrane M1 domain and extracellular E1 loop. The three domains are key components in GJ pore formation, as well as in voltage- and chemical-gating [e.g., by (Ca^2^^+^) changes] of GJs (Purnick et al., 2000; Sanchez and Verselis, 2014). The M1 domain also contributes to GJ voltage-gating polarity (Verselis et al., 1994), size selectivity, and unitary conductance (Kronengold et al., 2003). Importantly, the E1 domain constitutes the bulk of the aqueous pore and the E2 loop determines Cx compatibility in the assembly of heterotypic GJs, which are essential for GJ functions in cochlea and other organs (Sanchez and Verselis, 2014; Bai et al., 2018).

### Cellular Expression Patterns of Various Subtypes of GJs in the Cochlea

Multiple subtypes of Cxs are found in the cochlea, and many exhibit temporal regulation of expression during development, starting from early embryonic stages. For instance, Cx31 is expressed starting at E12, Cx26 at E14.5, Cx30 at E15, Cx43 at E15.5, and Cx45 at E17.5 (Lautermann et al., 1998; Xia et al., 2000; López-Bigas et al., 2002; Ahmad et al., 2003; Cohen-Salmon et al., 2004; Sun et al., 2005). Given GJs play a vital role in embryonic stages, it is necessary to investigate the cellular pattern and developmental time course of Cx expression in order to better understand the molecular basis of hereditary deafness. One of the earliest studies utilized a cDNA dot-blot analysis method, to quantitatively investigate the expression levels of 15 Cx genes in the mouse cochlea (Ahmad et al., 2003). The expression patterns and relative abundance of four major cochlear Cxs were determined by this method (from highest to lowest expression: *Gjb2*>*Gjc3*>*Gjb6*≈*Gja1*). Multiple studies have demonstrated that major subtypes of Cxs expressed in the cochlea are Cx26 (Kikuchi et al., 1995; Lautermann et al., 1998; Forge et al., 1999; Chang et al., 2008) and Cx30 (Xia et al., 2001; Ahmad et al., 2003; Teubner et al., 2003) in non-sensory cells of cochlea [e.g., supporting cells, lateral wall fibrocytes and basal cells of stria vascularis (SV)]. The study by Tang et al. (2006) was the first to show the expression of Cx29 in the cochlear Schwann cells. Other studies have shown that Cx43 is expressed in the SV and mature bony capsule (Suzuki et al., 2003; Cohen-Salmon et al., 2004), and Cx45 is found in the cochlear blood vessels (Cohen-Salmon et al., 2004). Other reported but more controversial Cxs appear to be minor cochlear Cxs, including Cx23 (Locher et al., 2015), Cx31 (Xia et al., 2000), Cx30.2 (Buniello et al., 2004), Cx31.1 (Buniello et al., 2004), Cx32 (López-Bigas et al., 2002), Cx36 (Liu W. et al., 2009), Cx40 (Buniello et al., 2004), and Cx59 (Buniello et al., 2004). Expressions of pannexin subtypes 1, 2, and 3 (Wang X. H. et al., 2009) have also been reported, but their ability to form GJ channels is controversial (Sahu et al., 2014). Data support that it is unlikely that they are assembled into cochlear GJs, but may function as hemichannels (Penuela et al., 2007).

The compatible pairs of major GJ-forming Cxs in the cochlea are Cx26–Cx30 (Ahmad et al., 2003), and Cx26–Cx31 (Liu X. Z. et al., 2009). Depending on the Cx composition, heteromerically and heterotypically assembled GJs demonstrate characteristic unitary conductance, permeability, voltage- and chemical-gating properties that are suited for their specific physiological roles (White and Bruzzone, 1996). It has been shown that heteromeric GJ channels assembled from Cx26 and Cx30 facilitate intracellular Ca^2+^ signaling twice as fast as their homomeric counterparts (Sun et al., 2005). In addition, the permeability of hetero- or homomeric GJs constituting Cx26 and Cx30 are found to display different charge- and size-selective properties (Sun et al., 2005). The following sections provide more detailed information about molecular structure of cochlear GJs and their constituting Cxs.

#### Cx26 and Cx30

GJs constituting Cx26 and Cx30 are the predominant GJs in the cochlea. These GJs connect all types of non-sensory cells in the organ of Corti, the connective tissue fibrocytes in the lateral wall and at least the basal cells in the SV (the expression of Cxs in the intermediate cells in the SV is controversial; Forge et al., 2003; Sun et al., 2005; Zhao and Yu, 2006; Liu W. et al., 2009; Kamiya et al., 2014). Given that Cx26 and Cx30 are not expressed in cochlear HCs (Ahmad et al., 2003; Zhao and Yu, 2006; Liu W. et al., 2009; Kamiya et al., 2014), it is generally believed that there are no direct intercellular conduits linking HCs and supporting cells. Cx26 expression and formation of Cx26-containing GJs are detected in the mouse cochlea as early as E14.5 (Sun et al., 2005; Kamiya et al., 2014). mRNA levels of both Cx26 and Cx30 peaks in the cochlea around P10, just before the onset of hearing in mice (Ahmad et al., 2003). In contrast, Western blots demonstrate that protein levels of both Cxs saturate around P15, and stay at the adult patterns and levels afterward. Cx26 and Cx30 exhibit gradient expression in the basilar membrane, with three-fold greater expression in the apex than the base (Zhao and Yu, 2006). In animals other than mice, immunofluorescent staining of guinea pig and rat cochleae shows an expression pattern of Cx26 and Cx30 consistent with that found in mice (Zhao and Yu, 2006; Liu and Zhao, 2008), namely that Cx26 and Cx30 are not expressed in the IHCs or OHC but in all types of cochlear non-sensory cells. However, largely due to limited availability of materials, the expression profiles of Cx26 and Cx30 in the human cochlea are still unclear. One preliminary study using human adult cochlea (Liu W. et al., 2009) reported that Cx26 and Cx30 are widely expressed in the lateral wall fibrocytes and supporting cells of Organ of Corti. A subsequent study by the same group showed that Cx26 and Cx30 proteins may not necessarily be co-assembled in the lateral wall of adult human cochlea, and homomeric GJs consisting of either Cx26 or Cx30 may be more prevalently assembled than previously thought (Liu et al., 2016). In human embryonic cochlea, expression of Cx26 has been detected as early as 11 weeks of gestation (W11; Kammen-Jolly et al., 2001; Locher et al., 2015). At W18, Cx26 is consistently detected in the supporting cells of the organ of Corti and the Kolliker’s organ, the outer sulcus cells, and Claudius cells (Locher et al., 2015). Cx30 is also detected in human embryologic cochlea in the Kolliker’s organ and the cells lining the outer sulcus cells (Locher et al., 2015).

Quantitative comparison of Cx26 and Cx30 protein levels indicates that Cx26 is expressed earlier than Cx30 in the cochlea (Sun et al., 2005), suggesting that loss of Cx26 expression may transiently result in a near elimination of GJs in the developing cochlea. Likewise, mice with a conditional knockout of Cx26 in the cochlea exhibit more severe and rapid cellular degeneration than mice lacking Cx30 (Sun et al., 2009). More importantly, hearing could be normal in the absence of Cx30 as long as Cx26 protein level is maintained at the WT level (Ahmad et al., 2007; Qu et al., 2012; Boulay et al., 2013; Chang et al., 2015a; Jagger and Forge, 2015). These observations suggest that the role of Cx26 in the developing cochlea is not replaceable by Cx30 (Ahmad et al., 2003; Qu et al., 2012; Boulay et al., 2013).

#### Cx29

In the mouse cochlea, Cx29 expression was first detected by the cDNA dot-blot hybridization and immunolabeling methods, and it is localized to the cochlear Schwann cells after birth, but not in the embryologic cochlea (Ahmad et al., 2003; Tang et al., 2006). These findings were supported by additional studies using both *in vitro* and *in vivo* studies (Eiberger et al., 2006; Li et al., 2007). Approximately 50% of Cx29^−/−^ mice exhibit early loss of high-frequency hearing and elevated sensitivity to noise damage (Tang et al., 2006), suggesting that Cx29 is required for normal cochlear function. Data from deaf patients have also linked mutations in Cx29 to hearing loss in humans, suggesting that Cx29 is a new candidate for studying auditory neuropathy (Yang et al., 2007).

#### Cx31

The expression of Cx31 is detected by *in situ* hybridization as early as E12 in Reissner’s membrane, SV and spiral limbus, the supporting cells, and fibrocytes in the spiral ligament (Xia et al., 2000; López-Bigas et al., 2002). In contrast, Cx31 expression disappears postnatally in most cells. After P12, only the type II and IV fibrocytes are positive for immunolabeling of Cx31 (Xia et al., 2000). However, no labeling in the sensory epithelial cells has been observed at any developmental stage (Xia et al., 2000; López-Bigas et al., 2002). Thus, the precise pattern of Cx31 expression in the cochlea is still unclear. Mutations in the *GJB3* gene encoding Cx31 (e.g., in-frame 3 bp deletion: 423-425delATT) have been linked to hearing loss in Chinese families with recessive deafness (Xia et al., 1998; Liu X. Z. et al., 2009).

#### Cx32

Cx32 is reported to be expressed in the SV, spiral limbus, Reissner’s and basilar membranes, and spiral ganglion neurons (SGNs) as detected by *in situ* hybridization (López-Bigas et al., 2002). However, these results are inconsistent with other studies utilizing cDNA microarrays, immunolocalization, and western blotting (Forge et al., 1999; Ahmad et al., 2003). In particular, Forge et al. (2003) failed to detect Cx32 using both RT-PCR and Western blots. Overall, Cx32 appears to be a minor player among cochlear GJs, as no severe hearing loss is observed in Cx32 null mice (Scherer et al., 1998). Cx32 mutations (e.g., Va163Ile and Glu186Lys) have been linked to X-linked peripheral neuropathy (e.g., X-linked Charcot-Marie-Tooth disease), and deafness may indirectly manifest as one of many phenotypes in humans (Matsuyama et al., 2001).

#### Cx43

Cohen-Salmon et al. (2004) were the first to find Cx43 expression in the developing cochleae of mice. They reported that Cx43 expression starts as early as E15.5. Fibrocytes and the mesenchymal cells below the basilar membrane are labeled at E16.5. By P5, Cx43 expression is detected in fibrocytes of the spiral ligament, in the SV capillaries and mesenchymal cells lining the basilar membrane. From P8 onwards, the expression pattern of Cx43 changes to the bony layer of the otic capsule, and the expression level increases with maturation, which is consistent with findings obtained by cDNA microarrays (Ahmad et al., 2003). However, details of Cx43 cellular localization are still unclear (Lautermann et al., 1998; Liu et al., 2001; Suzuki et al., 2003). One consistent finding appears to locate the Cx43 to the capillaries of the SV in mice. In human fetal cochlea, Cx43 expression is detected by immunostaining in a subgroup of spiral ligament fibrocytes at W14. By W18, these cells are more clearly defined as type I fibrocytes (Locher et al., 2015). A missense mutation (e.g., 976C→T, Thr326Ser) in Cx43 has been linked to hereditary hearing loss in humans (Yang et al., 2007).

#### Cx45

Expression of Cx45 is detected in the mouse cochlea by E17.5, in fibrocytes of the spiral limbus and ligament, mesenchymal cells under the basilar membrane and lining the scala vestibule, and in capillary cells (Cohen-Salmon et al., 2004). At P1, SGNs are more intensely labeled compared to embryologic stage. After P8, Cx45 is detected mainly in the capillaries and mesenchymal cells lining the basilar membrane (Cohen-Salmon et al., 2004). However, other groups reported that Cx45 was not detectable by RT-PCR or by western-blot in mature mouse cochlea (Forge et al., 2003). To date, no pathogenic mutations have been identified in Cx45 (Ouyang et al., 2011).

#### Pannexins (Panxs)

Panx1 and 2 expressions were first reported by Tang et al. (2008) as early as E16.5 in the mouse cochlea. Western-blot and immunolabeling show that Panx1 is expressed in the inner and outer sulcus cell, the Claudius cells and the SGNs. In contrast, Panx2 is detected only in the soma and nerve fibers of SGNs (Tang et al., 2008). Other studies (Wang X. H. et al., 2009; Zhao et al., 2015; Zhao, 2016) reported labeling of Panx1 in the supporting cells of the organ of Corti, and the Panx2 in SV and SGNs, Panx3 was detected in cochlear bone. Only one case report suggested that a homozygous *PANX1* variant (c.650G→A) may be associated with sensorineural hearing loss (Shao et al., 2016).

## Properties and Proposed Functions of GJs in the Cochlea

GJ intercellular channels possess a relatively large pore size (10–15 Å; Wingard and Zhao, 2015; Zhu et al., 2015b) that allow the passage of ions, nucleotides, miRNA, second messengers and other small molecules up to 1.8 kDa (Neijssen et al., 2005). Gating of GJs may regulate permeability to both larger (e.g., via gating to stay at various incompletely closed sub-states; Hesketh et al., 2009) and small molecules (e.g., by completely closed GJs; Bukauskas et al., 2002). Based on the studies that electrical coupling was not significantly affected in many mutant GJs known to affect hearing in humans (Kameritsch et al., 2005; Zhang et al., 2005), regulation of GJ permeability for cell-signaling and biochemically-important molecules (e.g., glucose, cAMP, nitric oxide) has been considered as the major function of cochlear GJs. The importance of GJ-mediated biochemical coupling is further supported by studies demonstrating that Cx26 mutations linked to human deafness (e.g., V84L, V95M, A88S) specifically affect GJ-mediated biochemical coupling (Beltramello et al., 2005; Zhang et al., 2005). Based on current understanding about the molecular properties of gating, developmental and spatial expression patterns, and molecular composition of cochlear GJs, multiple theories/hypotheses have been proposed for their functions.

### K^+^ Recycling Theory: GJs Facilitate the Diffusion of K^+^ Away From the Base of Hair Cells

The scala media is filled with endolymph which contains a high concentration of K^+^ of ~150 mM extracellularly. Acoustic stimulation induces K^+^ ions to flow into HCs through mechanotransduction channels, causing K^+^ ions to accumulate at the base of HCs if not quickly removed. The K^+^ recycling theory proposes that K^+^ accumulated during auditory transduction is removed by passive diffusion through intercellular GJs in supporting cells surrounding the HCs. These K^+^ ions are then recycled back into the endolymph through the GJs in the spiral limbus as well as the GJs in the lateral wall. One fault of this theory is that it does not identify the nature of the driving force for making the upward turn along the proposed K^+^ recycling pathway. Furthermore, new results obtained by multiple groups (Sun et al., 2009; Mammano, 2011; Zhao, 2017) from conditional Cx26 and Cx30 knockout mice are inconsistent with the predictions of classical K^+^ recycling theory:

(a) OHC degeneration starts days before the degeneration of IHCs (Wang Y. et al., 2009). As IHCs are the true mechanotransducer cells that are innervated by 90–95% of type I afferent fibers, and would be expected to accumulate large amount of the extracellular K^+^ that needs to be removed, this observation is not compatible with that expected by the K^+^ recycling theory.
(b) Data clearly show that the major cochlear GJs constituted by Cx26 play an essential role in the structural development (e.g., the opening of the tunnel of Corti) and functional maturation (e.g., cessation of spontaneous depolarization activities) of the cochlea (Wang Y. et al., 2009). Abnormal GJ formation is also observed in embryonic cochlea (Kamiya et al., 2014) weeks before the establishment of high K^+^, EP, and onset of hearing in mice (Wang Y. et al., 2009; Qu et al., 2012; Chang et al., 2015a; Jagger and Forge, 2015; Zhao, 2017). The timing of these observed events would make the proposed K^+^ recycling unnecessary.
(c) Conditional knockout of Cx26 before early postnatal stages (e.g., before P4) results in severe hearing loss in mice (Wang Y. et al., 2009; Chang et al., 2015a). Many cellular defects in the developing cochleae are observed, such as abnormal ribbon synapses, spontaneous depolarizing activities, pruning of type I and type II fibers of SGNs (Chang et al., 2015a). In contrast, conditional null of Cx26 expression in the cochlea after P16 does not significantly affect normal hearing (Chang et al., 2015a). These observations are not compatible with K^+^ recycling theory.
(d) There are other GJ systems in the cochlea, but not in the proposed K^+^ recycling pathway (e.g., GJs assembled from Cx29, Cx43), that play important cochlear functions that are clearly not linked to K^+^ recycling at all.

### Initiation of the EP

The EP is generated by a complex regulation of K^+^ fluxes by ion channels, membrane transporters/co-transporters and GJs in all types of cells in the SV (Prazma, 1975; Nin et al., 2008). The EP starts to develop around P5 in mice and reaches maturity at P17–18 (Sadanaga and Morimitsu, 1995). Importantly, EP is never developed in *Gjb6^−/−^* mice, which lack Cx30, presumably due to disruption of the endothelial barrier in the SV (Teubner et al., 2003; Cohen-Salmon et al., 2007). Alternatively, the reduction of EP has been related to the absence of Cx30 and reduced Cx26 expression in the basal cells of the SV (Boulay et al., 2013; Chen et al., 2014; Mei et al., 2017). In contrast, conditional knockout of Cx26 in the mouse cochlea reduces but does not eliminate, positive EP (Chen et al., 2014; Mei et al., 2017; Mammano, 2018). However, this phenotype results may be due to incomplete elimination of Cx26 expression in basal cells and fibrocytes in the lateral wall in the conditional Cx26-null mouse model. These studies indicate that Cx30 is needed for the initiation of the EP, but may indirectly act by reducing Cx26 expression (Ahmad et al., 2007).

### Intercellular Biochemical Signaling That Plays Vital Roles in Development and Survival of Cochlear Cells

Cochlear development requires well-established intercellular communication to coordinate cellular proliferation and differentiation. Given that the sensory epithelium in the cochlea is an avascular organ, GJs are proposed to act as an extension of the microvasculature to facilitate the transfer of metabolically-important molecules in cochlear supporting cells (Zhang et al., 2005; Chang et al., 2008). GJ-facilitated intercellular transfer of nutrient and signaling molecules may, therefore, play essential roles in cellular homeostasis during development and maintenance of cochlear functions. At the early postnatal stage, multiple phenotypes including abnormal ribbon synapse development, spontaneous depolarizing activities, fine-tuning of the innervation of the HCs are observed (Chang et al., 2015a). Missense or null mutations in Cx26 disrupt GJ formation as early as E14.5 in mice (Kamiya et al., 2014). In Cx30-null mice, double-electrode patch clamp recordings show that the absence of Cx30 does not significantly change GJ conductance among cochlear supporting cells (Chang et al., 2008). Dye diffusion assays, however, show that the rate and extent of intercellular transfer of multiple fluorescent dyes including a non-metabolizable D-glucose analog (2-NBDG) are severely reduced. In addition to glucose transport, a lack of Cx26 or Cx30 expression disrupts intercellular transfer of miRNAs (e.g., microRNA-96), inositol 1,4,5-trisphosphate, cAMP/cGMP, and ATP (Beltramello et al., 2005; Wang Y. et al., 2009; Conte et al., 2013; Forge et al., 2013; Zhu et al., 2015b; Mammano, 2018), all of which are likely to play critical roles in the cochlear development.

### GJs Contribute to Active Cochlear Amplification

Disruption of GJ expression in supporting cells has been shown to change active cochlear mechanics (Zhu et al., 2013, 2015a). Targeted deletion of Cx26 specifically in the Deiter and outer pillar cells alters nonlinear capacitance of OHCs and reduces high frequency DPOAEs. Reduction in DPOAEs progressively extends to the middle and low frequency regions with aging of mice (Zong et al., 2017). The results suggest that Cx26-containing GJs in the supporting cells play important roles in active cochlear amplification (Zhu et al., 2013; Lukashkina et al., 2017).

### Functions of GJs Constituted by Cxs Other Than Cx26 and Cx30

Cx29 is found to be exclusively expressed in Schwann cells surrounding SGNs in the cochlea (Tang et al., 2006). Cx29 does not form GJ plaques or functional GJ channels (Ahn et al., 2008), it is therefore proposed to play a major function as a hemichannel (Ahn et al., 2008) or by its novel association with the voltage-dependent K^+^ channel (Kv1; Altevogt et al., 2002; Cisterna et al., 2019) in the innermost layer of myelin-facing membranes of SGNs, allowing glial uptake of K^+^ from the extracellular space between axon and the myelin. The *Gjc3^−/−^*(Cx29 knockout) mice show a delay in the maturation of hearing thresholds and an early loss of high-frequency sensitivities. In addition, a prolongation in latency and distortion in the wave I of the auditory brainstem responses and elevated sensitivity to noise damages are found. However, the morphology of sensory HCs and DPOAE that depend on the integrity of outer HCs are normal in *Gjc3*^−/−^ mice, indicating that the organ of Corti is not directly affected. The phenotypes in *Gjc3*^−/−^ mice are explained by proposing that Cx29 hemichannels provide a pathway for removing accumulated K^+^ in peri-axonal space during high-frequency firings of the auditory nerve (Tang et al., 2006; Kagiava et al., 2015). Cx43 is found mostly in the bony capsule of the mature inner ear and is believed to play a critical role in the maturation of the otic capsule (Cohen-Salmon et al., 2004). Cx45 has been proposed to be involved in the inner ear vascular functions (Cohen-Salmon et al., 2004). Panx1 deficiency has been reported to activate the Caspase-3 cell apoptotic pathway and cause cochlear cell degeneration (particularly HCs; Zhao et al., 2015), thereby suggesting that Panx1 deficiency may lead to hearing loss. However, other studies have found that Panx1 is dispensable for normal hearing functions in mice (Zorzi et al., 2017; Abitbol et al., 2019).

## Knowledge About Cochlear Cxs Affects Design and Implementation of Translational Studies of Cochlear Gene Therapy

According to the World Health Organization (WHO)^2^, hundreds of millions of people are affected by hearing-impairment (defined as >40 dB hearing loss in at least one ear). Population-based studies in Europe and North America have identified a prevalence of approximately 1/1,000 of children affected by hearing loss^3^. Early interventions have been found to be a key factor to improve speech and language acquisition skills in affected children (Chen and Oghalai, 2016; Abdurehim et al., 2017). For patients with severe to profound hearing loss, cochlear implantation is currently the best available treatment option. Meanwhile, biology-based treatments are explored by many labs around the world for their intrinsic advantages in terms of improved sound perception quality and costs of long-term usage. More than half of congenital hearing loss cases arise from genetic causes, most commonly due to mutations in *GJB2* and *GJB6*. The most common *GJB2* mutations are: 35delG for European populations, 167delT for the Ashkenazi Jewish and 235delC for Eastern Asian populations. All three mutations generate functional null of Cx26, theoretically give rise to effects similar to those observed in functional null mouse models (Denoyelle et al., 1998; Cohen-Salmon et al., 2002; Dere et al., 2003; Wang Y. et al., 2009). In addition to Cx26, mutations in Cx30, Cx31, and Cx29 are also reported to cause heritable hearing loss. In most cases, deafness is caused by recessive mutations in a single gene, though some special cases of digenic inheritance have been reported (Riazuddin et al., 2000; del Castillo et al., 2002; Liu X. Z. et al., 2009). Monogenic deafness is potentially amenable to treatment by gene replacement or supplementation therapies, typically by utilizing a viral vector to express a WT gene (Zhang et al., 2018).

However, there are many challenging hurdles that must be overcome before cochlear gene therapy can be applied to humans (Zhang et al., 2018), including therapeutic time window, safe vector delivery route, transfection efficacy, and the specificity of the target cells. To date, many viral vectors have been used for cochlear gene therapy in animal models, such as AAV, adenovirus, herpes simplex virus, lentivirus, Sendai virus (Kurioka et al., 2016). However, most viral vectors have low transfection efficiency in cochlear supporting cells (Kilpatrick et al., 2011; Kurioka et al., 2016; Shu et al., 2016). It is interesting that a new synthetic viral vector (AAV-ie) shows promising high transfection efficiency in all types of cochlear supporting cells even injected at the adult stage (Tan et al., 2019). The majority of studies have used AAVs and results suggest it is the most promising viral subtype to be used in the next translational stage. Optimization of viral subtypes and their modifications, surgery delivery method and delivery routes have been reviewed in many published articles (Sacheli et al., 2013; Zhang et al., 2018; Wang et al., 2019) and they are not the focus here. Mutations in a single gene can lead to both syndromic and non-syndromic hearing loss [e.g., in cases of *GJB2* (MIM 121011), *COL11A2* (MIM 120290), *MYH9* (MIM 160775), *MY07A* (MIM 601317), *PDS* (MIM 274600), *CDH23* (MIM 605516), and *WFS1* (MIM 606201)]. Different mutations in the same gene may cause either dominant or recessive forms of non-syndromic hearing loss [e.g., *GJB2* mutations may cause either DFNA3 (MIM 601544) or DFNB1A (MIM 220290)]. Treatment designs will need to be considerably different (Gao et al., 2018). The phenotypic diversity demonstrates how the type of mutation, the position of the mutation within the gene, and allelic combinations (i.e., compound heterozygosity) can affect the overall clinical presentation.

Studies conducted in mouse models show that null mutations in Cx26 predominantly affect the normal development of the sensory epithelium in the cochlea before the onset of hearing (Wang Y. et al., 2009; Chang et al., 2015a). Relatively few studies on the cochlear pathological changes induced by Cx26 or Cx30 mutations have been performed in human samples. In one case-report of a 41-year-old female with congenital severe hearing loss associated with the Cx26 35delG mutation, examination of the inner ear found near-total degeneration of cochlear HCs (Jun et al., 2000). The patient’s tectorial membrane was also abnormal, and there was mild vestibular hydrops. Importantly, there was no obvious neural degeneration in the cochlea. It is unclear whether these pathological changes in the inner ear started from birth or gradually developed. These findings from human samples are consistent with the massive cellular degeneration observed in the cochlear sensory epithelium of conditional *Gjb2^−/−^* mice after the onset of hearing, suggesting that any gene therapy attempts to correct Cx26 defects in adults is unlikely to be successful (Yu et al., 2014). Some studies have reported success in preventing hearing loss when treatment is performed at embryonic stages (e.g., at E11.5) in Cx30 KO mice (Miwa et al., 2013; Minoda et al., 2015). These findings suggest that the time window of cochlear gene therapy for *GJB2* mutations need to be carried out early (perhaps embryonically) in humans to restore normal hearing (Zhang et al., 2018). Any treatment after malformation of the cochlea and HC death would have a significantly diminished chance of success.

Compared to Cx26 mutations, Cx30-null mutations show a slower time course of cellular degeneration in the mouse cochlea (Sun et al., 2009). The time window of treating patients with Cx30-null mutations may extend to a postnatal stage. Interestingly, over-expression of Cx26 can completely restore hearing sensitivity and prevent hair cell death in Cx30-null mice (Ahmad et al., 2007; Boulay et al., 2013), indicating that up-regulation of Cx26 might be a novel therapeutic strategy to prevent and treat deafness caused by Cx30 mutations. Another interesting finding is that mice homozygous for the A88V mutation in Cx30 show an improved high-frequency hearing threshold, while low frequency hearing is moderately impaired (Bosen et al., 2014; Kelly et al., 2019). This unusual finding may suggest a novel approach in clinical intervention using genome editing for treating high frequency hearing loss. Table 1 lists examples of Cx26 and Cx30 mutations that may demand different treatment time window. The expected outcomes of cochlear gene therapy based on our current knowledge learned from mouse models are also given.

**Table 1:** Types of Connexin26 (Cx26) and Cx30 human mutations and their possible treatment windows and outcomes by cochlear gene therapy.

Type of hearing loss	Mutation examples	Possible time window of treatment	Possible outcome
Late onset of mild hearing loss	Cx26: V37I, M34T, C202F, etc., Cx30: M203V, etc.,	Broad time window, possibly even after onset of hearing loss.	Complete recovery.
Syndromic hearing loss	Cx26: D66H, Y65H, G59A, G45E, D50N, H73R and N54K, etc., Cx30: G11R, A88V, etc.,	Embryonic (before W20)/early postnatal, likely need to be before the manifestation of hearing loss.	Uncertain treatment efficacy based on mouse model studies.
Non-syndromic hearing loss	Cx26: 35delG, 167delT, W44S, R75W, and R75Q, etc., Cx30: T5M, 342-KB DEL, etc.,	Embryonic (before W20)/Postnatal treatment needed.	Partial recovery of hearing. However, treatment benefit vs. risk is debatable.

Other factors that need to be considered in designing cochlear gene therapy for Cx-linked deafness should include the following: (1) Turnover rate of Cxs. Many Cxs, including Cx26, exhibit limited stability and degrade within 1–5 h (Fallon and Goodenough, 1981; Kelly et al., 2015). Cx30 exhibits a longer half-life of about 12 h (Kelly et al., 2015). It is unclear how over-expression of Cxs from viral vectors will affect this turnover rate, though such a strategy could still potentially alter GJ function and offer long-term treatment benefits; (2) Efficient delivery to the targeted cochlear cells. Studies have shown that ectopic Cx26 expression usually does not result in the formation of GJs in the cell membrane (Yu et al., 2014). Although it is unclear whether these intracellular or hemichannel Cx proteins are functional, the observation that the ectopically-expressed Cx26 in the cochlea of WT mice did not affect normal hearing (Yu et al., 2014) suggests that ectopically-expressed Cx26 does not seem to have harmful effects for the organ of Corti; and (3) A clinically-important question is how to maintain a long-term treatment efficacy of cochlear gene therapy. Many studies have shown that AAV1-mediated improvement of hearing is not necessarily long-lasting (Chang et al., 2015b; Kim et al., 2016; Isgrig et al., 2017). Therefore, more studies are needed before cochlear gene therapy can become clinically appealing.

Our current knowledge about GJ structure and functions in the cochlea indicates that Cxs play essential roles in the structure and functional maturation of the cochlea. Both Cx26 and Cx30 play vital roles in EP generation and maintenance. Reduced Cx26 expression is the likely cause of deafness even in cases of Cx30-null mutations since Cx26 expression at WT levels is sufficient for normal GJ function in the cochlea and the preservation of hearing. Before we can make a conclusion, one needs to keep in mind that studies have also shown that results obtained from mouse models may not necessarily be extrapolated to humans (and vice versa). Thus, larger animal models such as pig or non-human primates may be needed for further studies. Many human *GJB2* point mutations (e.g., M34T, V37I, L90P) give only mild-to-moderate hearing loss (Norris et al., 2006; Minami et al., 2013), which is very different from hearing phenotypes found in knockout mice. These studies warn us of the danger of oversimplification. We, therefore, need to be cautious in reaching any conclusion before we can get more accurate genotype and phenotype information from human samples (e.g., high-quality cochlear histopathology from genotype-confirmed patient inner ear samples) and non-human primate studies. Bear these in mind, studies thus far from the mouse models (mainly based on results obtained from conditional *Gjb2*-null mice) have indicated that the appropriate time window of gene therapy of Cx26 seems to be in early development, possibly necessitating surgery in human embryos. The ramifications of such aggressive intervention will need to be carefully analyzed.

## Author Contributions

XW and XL designed this article and generated Figure 1 and Table 1. WZ and YL made substantial contributions to the generation of Table 1.

## Conflict of Interest

The authors declare that the research was conducted in the absence of any commercial or financial relationships that could be construed as a potential conflict of interest.

## Footnotes

^1^http://davinci.crg.es/deafness/

^2^https://www.who.int/news-room/fact-sheets/detail/deafness-and-hearing-loss

^3^https://www.cdc.gov/ncbddd/hearingloss/data.html
